# Blue-LED-Light Photobiomodulation of Inflammatory Responses and New Tissue Formation in Mouse-Skin Wounds

**DOI:** 10.3390/life12101564

**Published:** 2022-10-09

**Authors:** Giada Magni, Francesca Tatini, Gaetano De Siena, Francesco S. Pavone, Domenico Alfieri, Riccardo Cicchi, Michele Rossi, Nicoletta Murciano, Gaia Paroli, Clarice Vannucci, Ginevra Sistri, Roberto Pini, Stefano Bacci, Francesca Rossi

**Affiliations:** 1 Istituto di Fisica Applicata “Nello Carrara”, Consiglio Nazionale delle Ricerche (CNR-IFAC), 50019 Sesto Fiorentino, Florence, Italy; 2 Department of Physics, University of Florence, 50019 Sesto Fiorentino, Florence, Italy; 3 European Laboratory for NonLinear Spectroscopy (LENS), University of Florence, 50019 Sesto Fiorentino, Florence, Italy; 4 EmoLED s.r.l., 50019 Sesto Fiorentino, Florence, Italy; 5 Istituto Nazionale di Ottica, Consiglio Nazionale delle Ricerche (CNR-INO), 50125 Sesto Fiorentino, Florence, Italy; 6 Department of Biology, University of Florence, 50019 Sesto Fiorentino, Florence, Italy

**Keywords:** photobiomodulation, blue LED light, wound healing, inflammatory response, collagen

## Abstract

**Background:** Recent studies evidence that blue-LED-light irradiation can modulate cell responses in the wound healing process within 24 h from treatment. This study aims to investigate blue-light (410–430 nm) photobiomodulation used in a murine wound model within six days post-treatment. **Methods:** A superficial wound was made in 30 CD1 male mice. The injuries were treated with a blue LED light (20.6 J/cm^2^), and biopsies were collected at 24, 72, and 144 h. Histology, fluorescence analysis, and advanced microscopy techniques were used. **Results:** We can observe an increase in the cellular infiltrate response, and in mast-cell density and their degranulation index correlated to the expression of the major histocompatibility complex after 24 h. Furthermore, after six days, the vessel density increases with the expression of the platelet-derived growth factor in the mast cells. Finally, collagen deposition and morphology in the treated wounds appear more similar to unwounded skin. **Conclusions:** Blue-light photobiomodulation stimulates several cellular processes that are finely coordinated by mast cells, leading to more rapid wound healing and a better-recovered skin morphology.

## 1. Introduction

Wound healing is an intricate and orchestrated process responsible for repairing disrupted tissues. In humans, physiological cutaneous healing has been described in three overlapping steps: inflammation, proliferation (or new tissue regeneration), and tissue remodeling [1]. Unfortunately, in susceptible subjects (e.g., patients with comorbidities or genetic predispositions), the healing process fails, the delicate balance between the loss of and new tissue formation is lacking, and the wound frequently becomes a chronic lesion [2,3,4,5]. From a biological point of view, wound healing involves a complex interaction between several cell types, including inflammatory cells, epidermal and dermal cells, the extracellular matrix, blood vessels, and plasma-derived proteins, coordinated by an array of cytokines and growth factors [1]. 

Photobiomodulation therapy (PBMT) refers to the biological impacts caused by applying light in the 400–1100 nm wavelength range [4,5,6]. Increasing evidence suggests that blue light can reduce tissue inflammation, promotes wound healing [7], and results in bacterial growth [8]. Blue-light photobiomodulation triggers a cascade of events attributable to the absorption of photons by intracellular photo-acceptors [9,10]. Among these effects, the impact of light on cytochrome-C oxidase can be observed: it induces an increase in cell proliferation, migration and differentiation, cytokine modulation, growth factor synthesis, and anti-inflammatory effects, thus stimulating the improvement of the healing process [7,9,10,11,12,13]. 

Our previous studies conducted on acute wounds [14] demonstrate that blue-light photobiomodulation (410–430 nm, 20.6 J/cm^2^) increases mast-cell (MC) degranulation and modulates cytokines release (e.g., tumor necrosis factor alpha, TNF-α, and transforming growth factor beta, TGF-β) [15] within 24 h from treatment. These effects lead to (i) a rapid reorganization of the inflammatory infiltrate; (ii) the stimulation of the angiogenesis process in a TNF-α-mediated fashion; and (iii) the migration of antigen-presenting cells, such as macrophages or myeloid dendritic cells. Moreover, TGF-β easily supports the differentiation of macrophages towards the M2 phenotype and the activation of plasmacytoid dendritic cells, which interact with T cells, thus promoting tolerance [16]. 

To further study the photobiomodulation effects of blue light on the wound healing process, this study aims to understand the response of cellular infiltrates, the angiogenetic process, and tissue regeneration within six days from treatment in an acute wound model. 

## 2. Materials and Methods

### 2.1. Animal Model and Blue LED Light Treatment

A total of 30 3-week-old CD1 male mice (Envigo, Udine, Italy), weighing between 15 and 20 g, were used in the experiment. The European Community guidelines for animal care (86/609/EEC) were followed, and written consent was duly obtained from the Italian Ministry of Health (791/2016-PR). All the experiments were performed blind. The mice were randomized, weighed, and anaesthetized with ketamine and xylazine (80–100 mg/kg, 10 mg/kg i.p.) (Sigma-Aldrich, Milan, Italy). The dorsal skin was shaved, and two superficial wounds (1 cm in diameter) were produced with an abrasor (speed set at 200 rpm) equipped with sandpaper (KWH Mirka Ltd., Jeppo, Finland, 68 μm particle size) [16,17,18]. The procedure of wound infliction was stopped when the underlying layer of skin with its gasping and gushing capillaries was observed. The two wounds were created in the same animal: one in rostral and the other in caudal positions. The wounds were spatially defined and separated from each other. One wound was randomly selected to be irradiated. The other wound was covered during the use of the blue light to avoid being exposed to accidental irradiation. The untreated wound on each animal was considered the control tissue. Only one treatment was performed per animal. 

The blue light (emission ranging from 410 to 430 nm) was applied with the following treatment parameters: 20.6 J/cm^2^ energy density, 0.69 W/cm^2^ power density, and 30 s treatment time. The fiber tip was kept at a 5 mm distance from the tissue, in continuous slow motion, and the irradiated area measured 5 mm in radius. The device was a previously described prototype [16]: the intensity distribution of the light at the top of the wound was homogeneous, as from a top-hat intensity source. An infrared thermal camera (InfReC, Nippon Avionics Co., Ltd., Japan) was used to monitor the superficial temperature dynamics, thus avoiding accidental thermal damage. 

Following the sacrifice of the animals by CO_2_ inhalation, dorsal skin patches (1 cm in length) were immediately excised and frozen at −80 °C. The dorsal skin samples obtained from the never-injured animals were used to compare the re-epithelialization in wounded tissues with unwounded ones. For fluorescence studies, two groups were studied: (1) not-treated wound (NTW) and (2) treated wound (TW). The follow-up times were 0, 24, 72, and 144 h for histology and immunofluorescence analyses. 

### 2.2. Histology and Immunofluorescence Analysis

All the collected biopsies were immediately split in half. A portion was embedded in Tissue-Tek (Killik, Bio Optica, Milan), quick-frozen at −80 °C to be sectioned by cryo-stat (Thermo HM 525, Bio-Optica, Florence, Italy) when required. The other portion was fixed in formalin and included in paraffin for the histological analysis. Finally, these last sections were stained with hematoxylin and eosin to analyze the entire cellular infiltrate. For the immunofluorescence investigations, specific cell types and growth factors were revealed using corresponding antibodies [19,20,21,22,23,24,25], as illustrated in Table 1. All the antibodies were purchased from Sigma-Aldrich or AbCam (Milan, Italy) and used according to the manufacturer’s instructions. Appropriate fluorescein isothiocyanate-labeled polyclonal antibodies obtained from rabbits or mice (1:32; Sigma-Aldrich, Milan, Italy) were used as secondary materials. Primary antibodies were applied overnight at 4 °C; secondary antibodies for 2 h at 37 °C. The omission of the primary antibody and substitution with an irrelevant one were used as controls for the immunofluorescence reactions. The following combinations for labeling were applied: Avidin-*Bandeiraea simplicifolia*; Avidin-Major Histocompatibility Complex II (MHCII); Avidin-Platelet Derived Growth Factor (PDGF); and Heat Shock Protein47-Alpha Smooth Muscle Actin (HSP47-αSMA). Histological images were acquired using a microscope equipped with a tablet camera VisiCam TC10 (VWR International, Milan, Italy). Epifluorescence photos were captured by a Zeiss Axioskop microscope equipped with a digital camera (Zeiss, Jena, Germany) connected to a PC (ED, Rome, Italy) hosting the software Axiovision 4 (Zeiss, Jena, Germany). 

### 2.3. Morphometry

Cellular infiltration was graded on a 0–3 arbitrary scale for each biopsy site [16,17,19]; the wounds were digitally photographed at a 25× magnification rate for studying the epithelialization process. The maximum epidermal thickness was measured in 10 epidermal tracts per specimen [19]. Immunostained sections were singularly placed under a microscope, adjacent to one another with no overlapping. The fields of the dermis were photographed separately at a 40× magnification rate. Sections from each follow-up (one section per staining) were stained, and a minimum of 10 acquisitions were used for each sample. In all cases, the image analysis was performed using Image J software (Rasband, W.S., Image J, US National Institutes of Health, Bethesda, Maryland, USA, https://imagej.nih.gov/ij/, 1997–2018, accessed on 28 November 2018) [16,17,19,25]. 

### 2.4. Two-Photon Fluorescence and Second-Harmonic Generation Imaging

A custom-made multiphoton microscopy setup was used to obtain co-registered two-photon fluorescence (TPF) and second-harmonic-generation (SHG) images of thin formalin-fixed, paraffin-embedded, unlabeled tissue sections. The experimental setup consisted of a custom-built microscopic platform for multimodal non-linear imaging. A femtosecond-pulsed Ti: Sapphire laser (Mira900F, Coherent, Santa Clara, CA, USA) provided 140 fs pulses at 76 MHz. The emission was tuned to a wavelength of 840 nm for these measurements. A few optical elements placed on the optical table served for conditioning the laser beam before entering the microscope mounted on a vertical stainless-steel honeycomb breadboard. The laser beam was scanned with a galvanometric scanning head and focused with a 20X objective lens PlanApo (Nikon, Tokyo, Japan), 0.75 NA. Fluorescence and SHG were separated from the excitation light using a dichroic mirror 685DCXRU (Chroma Technology Corporation, Rockingham, VT, USA). The SHG and TPF signals were in turn split using a dichroic mirror and, respectively, filtered by a narrow (10 nm FWHM) band-pass filter HQ420BP (Chroma Technology Corporation, Rockingham, VT, USA) and a fluorescence filter FF01-510/84-25 (Semrock, Rochester, NY, USA), before being collected on a couple of photomultiplier tubes H7422 (Hamamatsu, Hamamatsu City, Japan). Polarization optical elements were inserted in the path to obtain circular polarization in the sample. Further details were described in the previous study [26].

### 2.5. Statistical Analysis

Variance analysis was performed by Bonferroni-corrected *t*-tests, Tukey’s honestly significant difference tests (HSD tests), or chi-squared tests. *p* < 0.05 was considered as statistically significant. StatView 512^+^ (Abacus Concepts, Berkeley, CA, USA) and StatPlus (AnalystSoft, Walnut, CA, USA) programs were used. Significant results of the variance analysis among all experimental groups were compared with controls by Student’s *t*-test for unpaired values with two tails at each time point, assuming *p* < 0.05 as significant. 

## 3. Results

### 3.1. Thermographic Observation and Examination of Wounds

**Figure 1:**
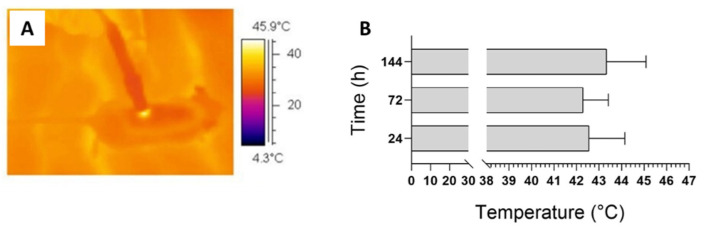
Infrared thermal recording during treatment (**A**). Average temperature reached in treated animals. Data are expressed as mean ± SD, *n* = 6 (**B**).

**Figure 2:**
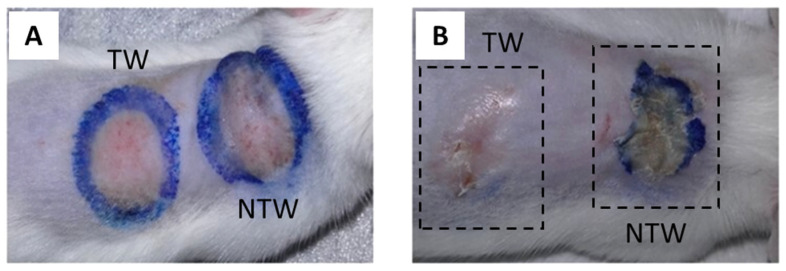
Dorsal skin abrasion 1 (**A**) and 6 (**B**) days after wound induction and treatment. TW: treated wound; NTW: not-treated wound.

Figure 1A presents an example of thermography performed during irradiation. The temperature never increased over 45 °C. The average value of the maximum temperature achieved during treatment is presented in Figure 1B: 42.55 ± 1.59 °C in the 24 h group; 42.28 ± 1.12 °C in the 48 h group, and 43.33 ± 1.75 °C in the 144 h group. The temperature dynamics presented a similar trend for all the animals: the maximum value was observed shortly after 1/2 the treatment time (approximately 18–22 s) and lasted for 30 s. When the application of the blue light was over, the temperature decreased to return to the initial values, corresponding to the body temperature of the animal. In addition, the image for day 1 shows that no macroscopic effect is visible yet (Figure 2A). In contrast, the same animal after 6 days experienced a clear improvement in the TW group’s appearance compared to the NTW group (Figure 2B). This last observation is also evidenced by the presence of the crust that is completely absent in the TW (Figure 2B).

### 3.2. Evaluation of Re-Epithelialization in Mice Model 

**Figure 3:**
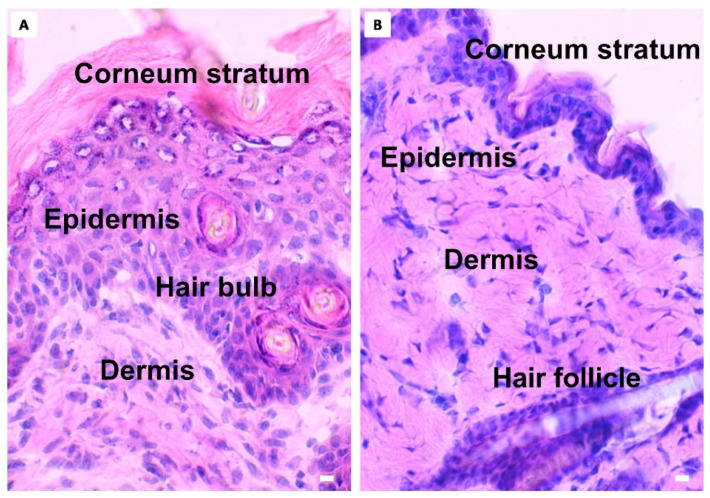
Microscopic aspects of NTW (**A**) and TW (**B**) groups. Differences in epidermal thickness observed in NTW group 144 h after the treatment. Light microscopy, hematoxylin–eosin. Scale bar: 10 μm.

The epidermal measurements of TW or NTW and control groups are depicted in Table 2. No epidermis was observed in the TW and NTW groups at 0 h after the induction of the wound. After 6 days from the treatment, only in the NTW group is the epidermal thickness different when compared to the control group (Figure 3A,B). This difference is due to the presence of a still thick corneum stratum (just below the crust), as observed in Figure 2B (in the NTW group) and 3A. In contrast, the TW group presents a thickness similar to the skin that was never injured, as can also be observed in Figure 2B (in the TW group) and Figure 3B.

### 3.3. Effects on Cellular Response

**Figure 4:**
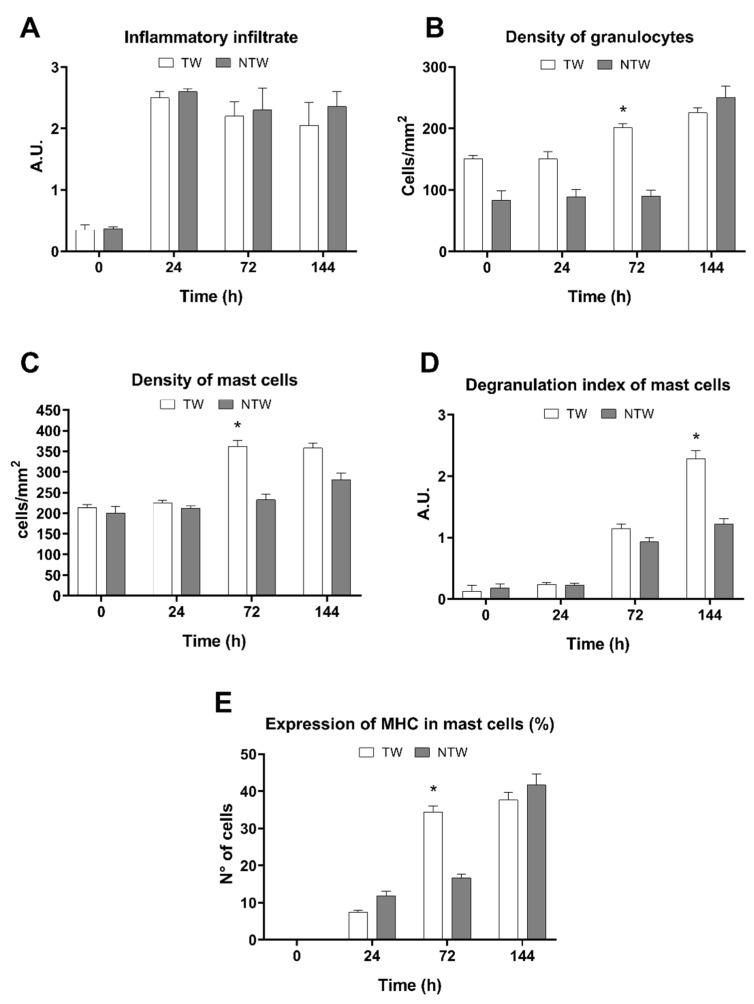
Evaluation of cellular responses in TW or NTW groups. (**A**) Cellular Infiltrate (unit of measure, a.u.: arbitrary unit). (**B**) Density of granulocytes (unit of measure: number of cells in tissue-section area). (**C**) Density of MCs (unit of measure: number of cells in tissue-section area). (**D**) Degranulation index of the MC (unit of measure, a.u.: arbitrary unit). (**E**) Percentage of the expression of MHC in MCs. White column: TW; gray column: NTW. Data are expressed as mean ± SD. Statistical analysis: * p < 0.05 vs. NTW at the same time point.

Significant differences can be observed in the cumulative inflammatory infiltrate at 0 and 24 h (Figure 4A). Nonetheless, the inflammatory infiltrate values were similar in the TW and NTW groups at 72 and 144 h (Figure 4A). However, there was a modu-lation of the cell populations involved in the wound healing process. Indeed, an in-crease in neutrophils (significant at 72 h) (Figure 4B), MCs (significant at 72 h) (Figure 4C), and their degranulation indexes (significant at 6 days) (Figure 4D) can be ob-served. Moreover, the MHCII expression in MCs was observed in the TW group by the first day following treatment, with a significant increase only on the third day (Figure 4E). 

**Figure 5:**
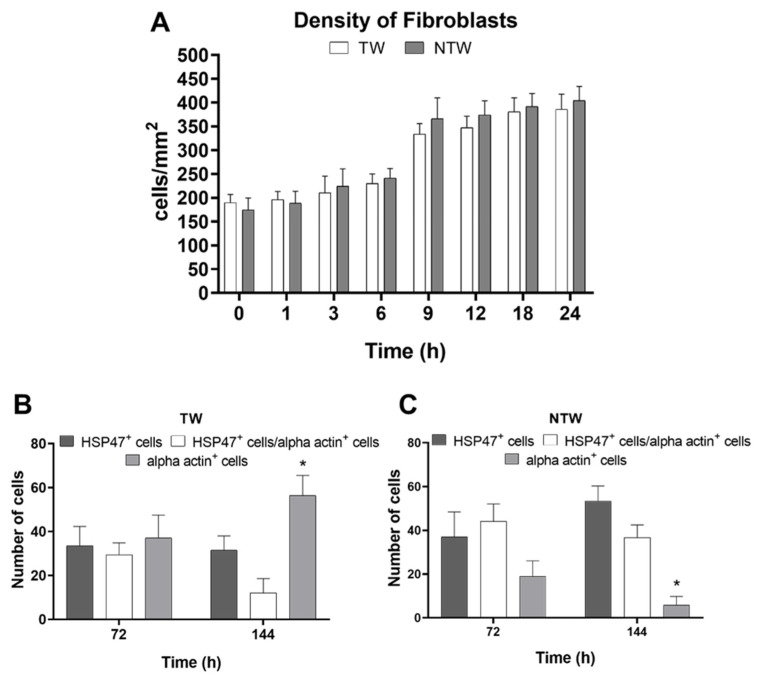
Immunofluorescence analysis of the behavior of fibroblasts and myofibroblasts. (**A**) Trend of the density of fibroblasts in the first 24 h (unit of measure: number of cells for tissue-section area) (**A**). Percentage of differentiation of fibroblasts (HSP47^+^) or myofibroblasts (alpha actin^+^). Black column: HSP47^+^ cells; gray column: alpha actin^+^ cells; white column: HSP47^+^/alpha actin^+^ cells. Data are expressed as means (**A**) or percent of cells (**B**,**C**) ± SD; significant *p*-values: * *p* < 0.05 vs. HSP47^+^ cells in TW and vs. HSP47^+^/alpha actin^+^ cells at the same time point.

Finally, earlier than 24 h, fibroblasts did not vary in density, as shown in Figure 5A. However, the density of the different populations of fibroblasts in the following days did vary in response to the blue-LED-light stimuli. In particular, fibroblasts and myofibroblasts presented a differential trend (Figure 5B,C). Indeed, the myofibroblasts began to differentiate in subsequent days, in both the TW and NTW groups. However, the percentage of intermediate forms appeared to be higher in the TW group and became significant at 6 days (Figure 5B,C).

### 3.4. Effects on Blood Vessels

**Figure 6:**
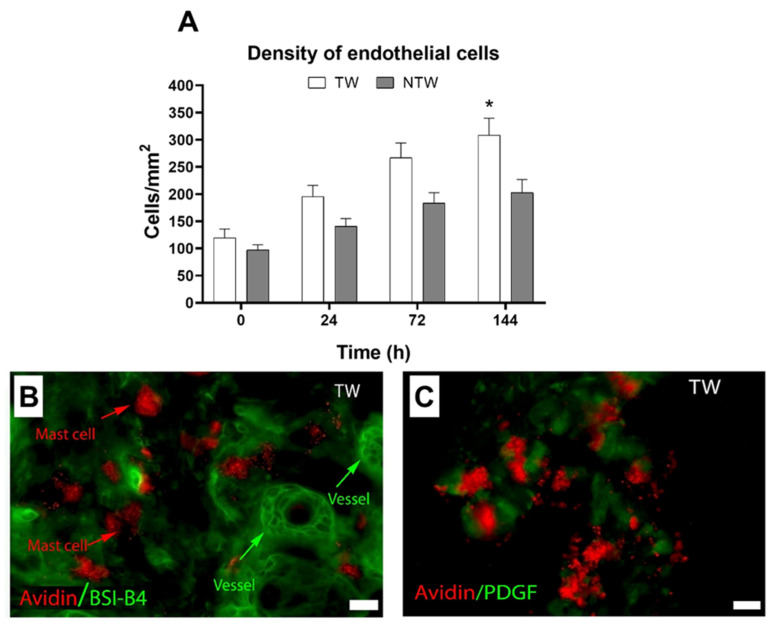
Histology and immunofluorescence analyses of angiogenesis in TW or NTW groups. (**A**) Density of vessels in TW or NTW groups (unit of measure: number of cells for tissue-section area), *p* < 0.05. (**B**) MCs and vessels in TW group at 6 days. (**C**) Expression of PDGF in MC granules at 6 days of treatment. White column: TW; black column: NTW. Scale bar: 10 μm; red: avidin; green: BSI-B4 (*Bandeiraea simplicifolia*) (**B**) or PDGF (**C**). Data are expressed as the mean ± SD. Significant *p*-values: * *p* < 0.05 vs. NTW at the same time point.

At 6 days, the density of the vessels reached the maximum peak, and the values were significantly different from those in the NTW group (Figure 6A). At the same time, in the TW group, the immunofluorescence analysis presented an increase in both cellular interactions between MCs and vessels (Figure 6B) and PDGF expression into MC granules (Figure 6C).

### 3.5. Effects on Collagen Deposition 

**Figure 7:**
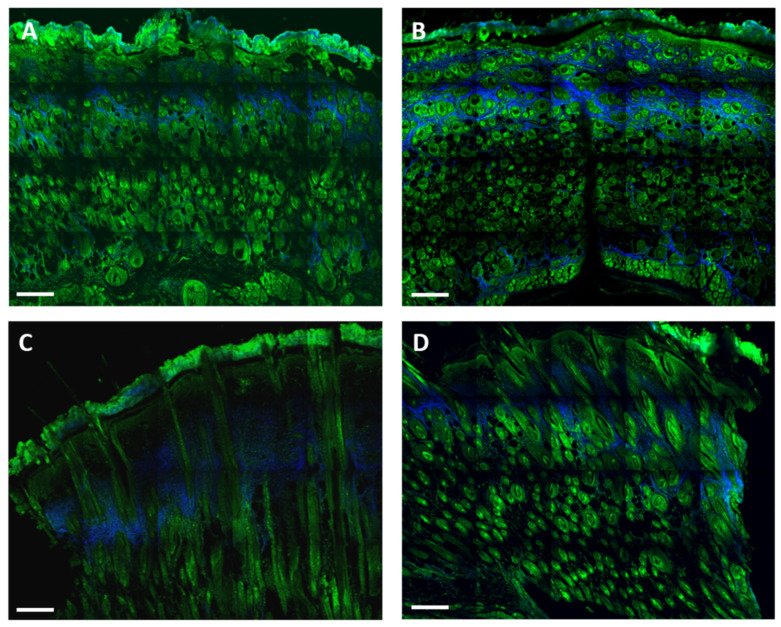
Merged TPF (green) and SHG (blue—collagen) images obtained from formalin-fixed, paraffin-embedded, unlabeled thin-tissue slices using multiphoton microscopy. The TW group at 3 (**A**) and 6 (**C**) days after treatment. The NTW group at 3 (**B**) and 6 (**D**) days. Scale bar: 200 μm.

By TPF and SHG (Figure 7), we observed that, three days after the treatment, the quantity of collagen was much higher in the NTW group with respect to the TW group (Figure 7A,B). The collagen distribution was observed to be sparse in the NTW group, whereas it was more uniform in the TW group. Six days following the treatment, the collagen amount and distribution were similar in the TW and NTW groups. (Figure 7C,D).

## 4. Discussion

In the current study, a blue light ranging from 410 to 430 nm was used. Its beneficial effects on the wound healing process were clinically demonstrated [28,29,30,31]. Our previous experiments studied the effects followed by the blue-LED-light treatment after 24 h from irradiation [16]. Here, we characterized different cellular responses evoked by blue-light irradiation until 6 days from exposure. 

A macroscopic qualitative analysis of the healing process in blue-light TW and NTW mice model groups was performed, and no adverse reactions were observed during and after treatment. During irradiation, the thermal analysis proved that the blue light induced a limited and controlled temperature increase in bleeding wounds due to predominant absorption occurring in the blood content (Figure 1). This mild photothermal stimulus can affect the healing process [14,32], as the temperature increase can modify the mechanical characteristics of the extracellular matrix and cell membrane permeability. These results are in agreement with our previous findings for a rat model [19]. 

The epithelialization measures show that the TW epidermis thickness was close to the controls (unwounded mice) one week after the treatment. At the same time, the re-epithelialization was still incomplete in the NTW group, as demonstrated by TPF-SHG microscopy, where the neo-formed tissue in the TW group presented more similarities with a control skin than an NTW. These results appear contradictory compared to a previous model that showed a lack of stratum corneum in untreated skin [19], but are in agreement with others [27,33]. However, the mechanisms of re-epithelialization under the effect of blue light are still controversial and often debated: the experiments of Denzinger et al. [34] about peripheral blood mononuclear cells show that the immunomodulating properties of blue light do not affect re-epithelialization in cultured keratinocytes. 

The increase in the cellular infiltrate in the TW group, revealed by histological analysis, may be considered as an early inflammatory response stimulated by blue light [16] (Figure 3A,B). Indeed, the evidence that at 6 days in the NTW group the cellular infiltrate is still high compared to the TW group, supports this hypothesis (Figure 3A,B). Furthermore, the increase in the TWs of the MC density at 72 h (Figure 3C) and the degranulation index at 6 days (Figure 3D) confirms that these cellular types play a crucial role in wound healing [35]. The selective and localized temperature increased, resulting from the photothermal effect stimulated by blue-light irradiation, leading to MC activation. This phenomenon is likely due to specific receptors also expressed in mice [36]. Following the activation, MCs released various cytokines that stimulate other cell types and, together with PDGF (Figure 6B,C), facilitate the angiogenetic process [37,38]. As shown in our results, at 6 days, the density of the vessels reach the maximum peak (Figure 6A), which is significantly different compared to NTWs. Moreover, the MHC expression of MCs was significant in the TW group three days following the treatment (Figure 4E). This last result suggests that MCs might be involved in the T-cell response also occurring in wound healing [35,36,37,38,39].

Finally, as demonstrated by HSP47 expression, fibroblast activation (Figure 5A) is critically dependent on the inflammatory environment [40]. In particular, macrophages and MCs mainly affect the behavior of fibroblasts and phenotypes by producing soluble paracrine factors, including PDGF, VEGF, and FGF [35,40]. This hypothesis was confirmed by the fact that myofibroblasts began to differentiate in TW and NTW groups, and adopted significant values at 6 days only in the TW group. The blue LED light seems to have had an effect on collagen deposition documented by an increase in the amount of collagen in the TW group with respect to the NTWs at three days. These results are coherent with the increase in the myofibroblast cells in the TW group, while the intermediate forms in the NTW group are lower. These differences are reinforced at 6 days (Figure 7C,D). 

## 5. Conclusions

This study examined the effect of blue-light treatment on several cell types involved in healing and collagen deposition in acute wounds created in murine models. Our results suggest that after six days, photobiomodulation with blue light evokes an MC response that establishes an accurate and finely coordinated cellular response. These cells stimulated (i) an early inflammatory response; (ii) angiogenesis, thanks to the secretion of TNF-α [36]; and (iii) the response of fibroblasts that switched into myofibroblasts, which played a role in wound closure, evidenced by re-epithelialization and collagen deposition [36,37,38,39,40,41]. Therefore, together with our previous investigation [15,16,19,41,42,43], these results show that blue-LED-light irradiation induces a quicker healing process.

## Figures and Tables

**Table 1:** Antibodies used in immunofluorescence studies.

Target	Antibody	Dilution
Myofibroblasts	Anti-alpha-SMA	1:50
Fibroblasts	Anti-HSP47	1:50
Mast Cells	Avidin	1:400
Vessels	*Bandeiraea simplicifolia* (*Griffonia simplicifolia*)	1:10
Vessels	Anti-PDGF	1:50
Granulocytes	Anti-Ly6G	1:50
Dendritic Cells	Anti-MHCII	1:50

**Table 2:** Different values of epidermal thickness in TW or NTW groups. Unwounded skin: skin never injured; ref: reference in the literature [19,27]; 0: time immediately after the induction of the skin wound; SE: standard error; *p*-values of TW and NTW groups calculated vs. CTRL.

Time (h)	Type of Wound	Counts (μm) Mean ± SE	*p*-Value
-	Unwounded skin	30 ± 2.6 (ref)	-
0	NTW	10.1 ± 0.50	<0.05
0	TW	11.8 ± 1.38	<0.05
24	NTW	31.1 ± 1.44	Not significant
24	TW	30.0 ± 2.58	Not significant
72	NTW	36.7 ± 0.48	Not significant
72	TW	32.7 ± 0.50	Not significant
144	NTW	49.64 ± 2.24	<0.05
144	TW	33.0 ± 6.25	Not significant

## Data Availability

The data presented in this study are available at the request of the corresponding author. Data are not available to the public due to ongoing study.
